# The Home Treatment of Consumption

**Published:** 1910-02-05

**Authors:** J. E. Bullock


### The General Practitioner's Column.

[Contributions to this Column are invited, and, if accepted, will be paid for.]

### THE HOME TREATMENT OF CONSUMPTION.

By J. E. BULLOCK, M.D.

If a consumptive has to carry out treatment at home it will be of great advantage, if he has previously been in a sanatorium, he will then more readily understand how he ought to continue the treatment, and the experience gained will be of great use to him.*

Phthisis is a chronic disease and time must elapse before it can be thoroughly arrested. If treatment on sanatorium lines has been pursued for, say, four months, further treatment should be continued for quite twice that time, as, assuming that the patient has done well, he must gradually test whether he is able to resume his ordinary avocations. It may be suggested that it is difficult to carry out sanatorium treatment satisfactorily in an ordinary house, first because it is difficult to adapt it to the purpose, and, secondly, because the other inmates do not care to accommodate themselves to what is required. But if a patient has learnt the principles which govern sanatorium life, it will be quite possible for

* See the "Educational Influence of Sanatorium Treatment." The Hospital, June 19, 1909, p. 315.

him to carry them out at home, with a few modifications, and if others in the house wish him to get well they will readily accede to all that is necessary for him; moreover, it is only general hygiene, which is beneficial to all, that is required. There need not be any fear of infection from the patient, for with precautions quite easily carried out there is no danger whatever. One of the main difficulties in obtaining "plenty of air" in an ordinary dwelling-house is the objection raised by others on the score of draughts and coldness in winter. A "draught" is a great bugbear to most people and few will tolerate it. A narrow current of air, markedly colder than the surface of the body, is to be avoided by everyone, but if air is admitted to a room by a widely-open window, very little draught is felt; it is merely like feeling the air out of doors, and if too cold can be guarded against by warm clothing and artificial heat. It must be considered a general principle that the air of the rooms of a consumptive should approximate to the temperature of the outside air, except in winter; the temperature of a room may be as low as 50° F.

### Town Patients.

The treatment of a consumptive in a town should only be undertaken if necessity compels; the advantage of country over town is that the air is much purer and the house can be more favourably placed with respect to aspect and surroundings. The soil should be of a dry porous nature, the lower greensand or chalk being the best. Care must be taken that the house stands on the original subsoil and that "rubbish" of different kinds has not been thrown in to take the place of sand, which may have been removed for building purposes. It should also stand back from the road, so as to avoid dust, but the fear of dust should never cause the windows to be closed. For this reason the rooms occupied by the consumptive may preferably be at the back of the house and should, if possible, command an open space and pleasant view. They must be fully exposed to all the available sun, and it is an advantage if a room opens on to a balcony or verandah. Seeing that there must always be free ventilation through the rooms (by means of open windows and doors), they need not be especially large; 1,700 cubic feet, exclusive of space required for furniture, should be provided, and this can be obtained by a room 11 ft. high and about 13 ft. in length and breadth.

The floors when not of inlaid wood or of well-laid polished boards, should be covered with linoleum; there may be one or two rugs (of a material not causing "fluff") which can be daily taken up and shaken outside the house: a nailed down carpet cannot be allowed; the floor must be wiped over daily with a damp flannel fastened over an ordinary broom—a broom must not be used unless covered in this way, and the flannel must be steeped in water after use. The walls should be either painted or varnished so as to provide a smooth surface, which can be wiped over with a damp cloth; nothing should be placed on the walls, except a few pictures, which can be easily dusted; as far as possible ledges in the skirting boards and woodwork of the door and window-frames must be done away with by bevelled wood and plaster, filling up all crevices and giving a slanting surface; the top of the architrave of the door and the top of the window-frames which afford a lodgment for dust, should be covered with a bevelled strip of wood, giving an ornamental finish. In the same way the junction of the skirting boards and floor should be rounded off with wood.

Casement windows admit more air than sash windows in proportion to their size; either must open widely and must remain open in all weathers; if there is no verandah or overhanging eaves, some protection from heavy rain is necessary; wooden louvred shutters, folding back outwards when not in use, are applicable to sash windows, and an upper division opening outwards and hinged from the top should be over casement windows; the windows should open to as near the ceiling as possible; there must always be a current of air through the room, obtained without unpleasant draught by a free opening of the windows: when the wind is not blowing directly into the room, the door also must be placed more or less widely open; when the door has to be shut, through ventilation should be effected by a fanlight over the door. In order to ventilate a room thoroughly, it is necessary that the air be changed two or three times in an hour. When a patient has become thoroughly accustomed to air, he will not object to its blowing upon him as it passes from the window to the open door: the window and door ventilation should be continued day and night, and only reduced when the weather is very boisterous. In an ordinary house it is not usually possible to have the doors opening direct to the open air, so a window outside the room must be kept open.

### Minor Details of Room Furnishing.

It is preferable that there should be but little upholstery in the rooms, as such harbours dust. Comfortable chairs are made of polished wood, and may be of any elegant design so long as there are no crevices for the lodgment of dust: light and portable couches are made of wicker work; they should have a leg-rest quite distinct from the body of the couch; this is preferable to the arrangement with a leg-rest sliding under the seat, as less likely to harbour dust. Movable cushions with washable covers are permissible. There need be no shelves about the rooms. A bookcase should be closed with well-fitting glass doors; a nicely polished table will need no cover; all the furniture should be such as can be readily wiped over with a damp duster, and any which cannot be lifted should be on easy-running castors, so that if placed against a wall it can be readily moved out and dust behind and beneath it wiped up; washable curtains hanging from a brass rod are permissible. An adjustable frame covered with muslin can easily be placed in any window; it is effective as a curtain or screen; in the winter it moderates draught, and in summer nights serves to keep out moths, etc. The bedstead should be of iron, with a wire-wove spring mattress and covered with a hair mattress, sheets, and the necessary blankets; over all should be placed a light washable coverlet. It must not be placed in a corner of the room, but in a space where air can freely circulate all round it, and stand out about a foot from the wall; a screen may be placed between it and the window if there is too much direct draught. The windows must never be closed to warm the room (this being effected by artificial heat), though they may be closed while dressing or undressing in very cold weather. A hot-water bottle is much appreciated by those who suffer from cold feet. The patient should be in his day room as little as possible, and spend much time out of doors, either taking exercise or in some sheltered spot. In an ordinary house an open coal fire is the best form of heating; care must be taken that it does not produce dust and smoke; the fireplace is useful in assisting ventilation; slow-combustion stoves and oil lamps are not allowable, as they use up the air extravagantly, and are useless for ventilation. The lighting must be obtained at the least expenditure of the air of the room; for this purpose electric light is the best, and next to that candles or oil, and gas only if it cannot be avoided.

### Special Treatment—Hygienic.

In the matter of treatment, the patient has to carry out principles of hygiene in general and warfare against the tubercle bacillus in particular. In the latter connection the system must be braced up to resist the bacillus, and the bacillus must be destroyed outside the body; the latter is comparatively easy, as the bacillus is limited to the sputum. It has been satisfactorily proved that the ordinary expired air does not contain tubercle bacilli, but that infection is confined to the sputum, which may be ejected in fine spray by coughing, sneezing, and excited talking, or in a more solid state in the expectoration. When coughing, the patient should always hold a handkerchief before his mouth, and, of course, turn away from another person; it has been shown that in coughing tubercle bacilli may be carried three feet, or about an arm's length; thus careless coughing may be a source of spreading the disease. The danger from sputum arises when it is allowed to dry, and particles containing active bacilli are carried about as dust. The patient must carry in his pocket a special flask into which he can spit when walking about, and at other times have at hand a covered cup for the same purpose. The sputum may be emptied down the water-closet, taking care that none adheres to the top of the pan, or it may be burnt; it must *never* be emptied on a dust-heap, where it may dry and be disseminated. Both flask and cup must be emptied daily, well washed, and boiled for ten minutes in soda water. If the sputum is scanty it can be easily turned out if the flask is lined with butter-paper; if paper is not used, and the sputum adheres to the sides, a solution of lysol (1 per cent.) will render it more easily emptied; otherwise a small quantity of carbolic acid (1 in 20) may be placed in cup or flask. If at any expectoration the outside of the flask is soiled it should not be wiped with the handkerchief, but cleansed at once by boiling water poured over it; the hovering of flies round the cup indicates that the outside is not perfectly clean. The patient must not spit into his handkerchief; but as it has to be used to wipe the mouth after expectoration, and held before the mouth when coughing or sneezing, it should be placed at the end of each day in a solution of carbolic acid (1 in 50) preparatory to the usual washing. A clean handkerchief should be put under the pillow each night, and after it has been used once it should be placed in a receptacle and treated in the morning like the day handkerchief. I am not in favour of paper handkerchiefs, as they easily tear and are not so cleanly as the ordinary handkerchiefs, used as above directed, but paper serviettes may be used at meal-times.

### Diet.

To brace up the system the diet must be light and nutritious, and as a rule there should be three good meals a day. The exact dietary must be regulated by individual needs, and only an outline can be given here. Before rising in the morning the patient may take a glass of hot milk. Breakfast should consist of a varied menu, containing porridge with milk or cream, bacon, ham, eggs, or fish, etc., according to taste; tea or coffee, bread and plenty of butter, fruit when available. A glass of milk at 11 A.M. or after the morning exercise, which should terminate about 12, so that the patient can get an hour's rest before dinner. This meal may consist of soup or fish, any meat except pork or veal, no "made dishes"; game, poultry, etc.; vegetables of any kind, milk or suet puddings and stewed fruit, butter, cheese, and biscuits; at 4 to 5 P.M. a simple "afternoon tea." Exercise or recreation is taken during the afternoon, allowing an hour's rest before 7 P.M., when a meal similar to dinner may be taken, and the day ended with a glass of hot milk at bedtime. Milk should be taken at lunch and dinner instead of beer or wine. Dyspepsia can usually be avoided by exercise and dieting. Care must be taken that it is not promoted by over-feeding; constipation must be guarded against by mild aperients if necessary.

### Exercise.

The amount of exercise must be governed by the body temperature; if it is normal before breakfast exercise may be taken during the day. As the temperature usually rises after exercise and subsides with rest, there need be no alarm if after active exercise the temperature is found to be 100° F., provided that it comes down to normal after half an hour's rest; if the temperature remains elevated, further rest must be taken until it is normal. The exact nature and amount of exercise can only be decided upon by a knowledge of individual circumstances, and must be regulated by the doctor. The patient must consider the exercise excessive if he does not readily recover from healthy fatigue and the temperature remains above normal. Exercise may consist of walking, gardening, croquet, bowls, ring quoits, etc. With respect to indoor recreations, crowded places of assembly must be avoided on account of the impurity of the air. A consumptive should only go to a church or place of worship if he can choose a seat near an open door or window, with a clear space not in proximity to other people. Theatres, concert-halls, etc., he must scrupulously keep away from. Dancing is to be discouraged, as, besides leading to excessive exercise, it usually takes place in hot, crowded, and dusty rooms. Games of cards, draughts, chess, etc., are permissible; also music, except with wind instruments. Singing is allowable, unless the patient is subject to any laryngeal affection. Reading may be done while resting and on rainy days; it must not take the place of exercise. Much writing, painting, etc., must not be undertaken if it involves a crouched position. Outdoor bathing and swimming causes too much exercise; but the daily bath is necessary; whether it is hot, tepid, or cold must depend on the reacting powers of the individual and the time of year. If the patient perspires much a sponge bath is imperative. The body must be warmly clothed, so that the patient does not get chilled. The cellular (Aertex) underclothing is excellent; it is light, warm, absorbent of perspiration, allows free ventilation from the skin, and does not get spoilt in washing—in this respect surpassing woollen garments.

Marriage must be postponed until there is a certainty of the arrest of the disease. In the case of women a long period of arrest is necessary. Pregnancy and childbirth throw a great strain upon the system, and many instances are known of aggravation of the disease following childbirth.

For successful home treatment the patient must conscientiously follow the rules laid down for him by his doctor. The chest and general condition should be examined periodically, and advice taken as to future conduct. If there is favourable progress it will be sufficient if this is done once a week, the patient being careful to notify at once any abnormal symptom.

